# Rational Design of High-Performance Photocontrolled Molecular Switches Based on Chiroptical Dimethylcethrene: A Theoretical Study

**DOI:** 10.3390/molecules29204912

**Published:** 2024-10-17

**Authors:** Li Han, Mei Wang, Yifan Zhang, Bin Cui, Desheng Liu

**Affiliations:** 1 School of Physics, Shandong University, Jinan 250100, China; hehehanli@163.com; 2 School of Physics and Electronic Engineering, Jining University, Qufu 273155, China; yzhangevonne@163.com; 3 School of Science, Shandong Jiaotong University, Jinan 250357, China; wm@sdjtu.edu.cn

**Keywords:** photoswitch, first principle, charge transport, switching effect

## Abstract

The reversible photo-induced conformation transition of a single molecule with a [5]helicene backbone has garnered considerable interest in recent studies. Based on such a switching process, one can build molecular photo-driven switches for potential applications of nanoelectronics. But the achievement of high-performance reversible single-molecule photoswitches is still rare. Here, we theoretically propose a 13,14-dimethylcethrene switch whose photoisomerization between the ring-*closed* and ring-*open* forms can be triggered by ultraviolet (UV) and visible light irradiation. The electronic structure transitions and charge transport characteristics, concurrent with the photo-driven electrocyclization of the molecule, are calculated by the non-equilibrium Green’s function (NEGF) in combination with density functional theory (DFT). The electrical conductivity bears great diversity between the closed and open configurations, certifying the switching behavior and leading to a maximum *on–off* ratio of up to 10^3^, which is considerable in organic junctions. Further analysis confirms the evident switching behaviors affected by the molecule–electrode interfaces in molecular junctions. Our findings are helpful for the rational design of organic photoswitches at the single-molecule level based on cethrene and analogous organic molecules.

## 1. Introduction

Over the past few decades, the advancement of various sophisticated nanotechnologies has made it possible to investigate molecules at nano- and sub-nanometer scales [1,2,3]. Research on single-molecule multi-function nanodevices, e.g., rectification [4,5,6], switching [7,8,9], motor [10,11,12], and the gate effect [13,14], have been conducted, and a wide range of applications have been covered, including biosensors [15,16], logic circuits [17], drug release [18], and catalyses [19]. The rational design and fabrication of logical switches with ultra-high temporal–spatial resolution based on the photosensitive characteristics of organic molecules is one of the most popular research topics. Working as the controllable function unit of logical switches, the photo-induced switching progress of organic molecules, such as diarylethene [20], fulgides [21], azobenzene [22], and spiropyran [23], are inherently accompanied by transitions in geometrical and electronic structures, which are triggered by light of various wavelengths [24]. Recently, a novel molecular switch leveraging photoisomerization based on a [5]helicene backbone, cethrene, has been proposed and has received considerable attention [25,26,27].

Helicenes are chiral π-conjugated systems composed of benzene rings, with the synthesis of the first [5]helicene dating back over a century [28,29]. Helicene-based switches [30] exhibit tunable switching behavior in response to diverse external stimuli, including variations in electrical conductivity and magnetization induced by light, heat, and acids/bases [31,32]. The first photoswitch based on the helicene structure was developed in 1999, involving a reversible transition between the ring-*open* form and ring-*closed* form through carbon–carbon (C-C) bond formation and breaking in the fjord region [33]. In 2016, Juríček et al. reported the synthesis and properties of cethrene, a helical chiral molecule with a [5]helicene backbone, which could not be isolated as solid material owing to the oxidative dehydrogenation of the ring-*closed* isomer [25]. Their follow-up work, investigated a series of functionalized [5]helicene derivatives and found that the dimethyl derivative demonstrated the highest configurational stability [34]. Subsequently, a chiroptical photoswitch, 13,14-dimethylcethrene, that can be reversibly transformed via an electrocyclic reaction has been successfully synthesized and is believed to have the potential to be designed as a magnetic photoswitch. The ring-*closed* isomer has unprecedented stability, benefiting from two methyl substituents installed in the fjord region [26]. In 2022, Dumele et al. reported a photochemical magnetic switch based on 4,11-dioxo or 4,11-bis (dicyanomethylidenyl) substituted 1,14-dimethyl[5]helicene with bistable spin states [35]. In 2023, the first cethrene derivative showing enhanced stability in its ring-*open* form compared to its ring-*closed* form was synthesized [36]. Current research on helicene-based photoswitches predominantly centers on molecular properties—optical activity, stability, and switching dynamics—while their potential applications in nanodevices remain underexplored.

The sandwich structure, consisting of two electrodes separated by a functional molecule, is the standard device configuration for single-molecule-level photoswitches. Based on such configurations, the performance of photoswitches is influenced by multiple factors, such as electrode materials, molecular properties, electrode–molecule interface characteristics, and fabrication methods [37]. A critical challenge in creating photoswitches is achieving stable and controllable contact between the electrodes and molecules. The appropriate choice of anchoring groups that determine the interfacial coupling strength between the electrode and the molecule is essential for the reversible photoisomerization of the functional molecule [38]. The combination of thiol (–SH) anchors and Au electrodes has been extensively employed in both theoretical and experimental studies of molecular photoswitches. In 2003, Dulić et al. investigated the unidirectional switching behavior of diarylethene-based molecular junctions. The cyclization of the ring-*open* form was not detectable due to the quenching effect, and only the transformation from the ring-*closed* to ring-*open* form was observed [39,40,41]. To date, although completely reversible photoswitches based on diarylethene and other functional molecules have been successfully prepared, the mechanism of the quenching effect is still not fully understood [38,42,43]. Further exploration of the electrode–molecule interface characteristics that play a key role in the switching behavior of nanodevices is needed.

**Figure 1:**
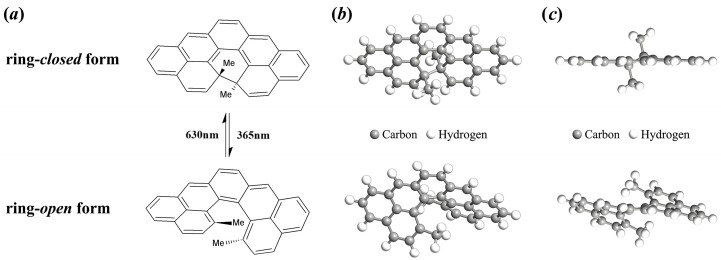
A schematic diagram of (**a**) the photoisomerization process of 13,14-dimethylcethrene and (**b**) the corresponding 45° top views and (**c**) side views.

In this work, we reported molecular photoswitches utilizing the 13,14-dimethylcethrene molecule and investigated the effect of molecule–electrode interfaces on the electronic transport property of devices at the single-molecule level. As shown in Figure 1, the light-driven electrocyclization of 13,14-dimethylcethrene from the ring-*open* form to the ring-*closed* form can be triggered by using visible light (630 nm) or heat, and reversion to the ring-*open* form occurs upon exposure to ultraviolet (UV) light (365 nm). We designed Au–molecule–Au junctions based on dimethylcethrene, and the simulation of electronic transport properties was performed using advanced calculations that combine the non-equilibrium Green’s function (NEGF) method with density functional theory (DFT) [44]. The numerical results show that the maximum *on–off* ratio of these molecular junctions can reach as high as 10^3^.

## 2. Results and Discussion

### 2.1. I–V Characteristics and On–Off Ratio

**Figure 2:**
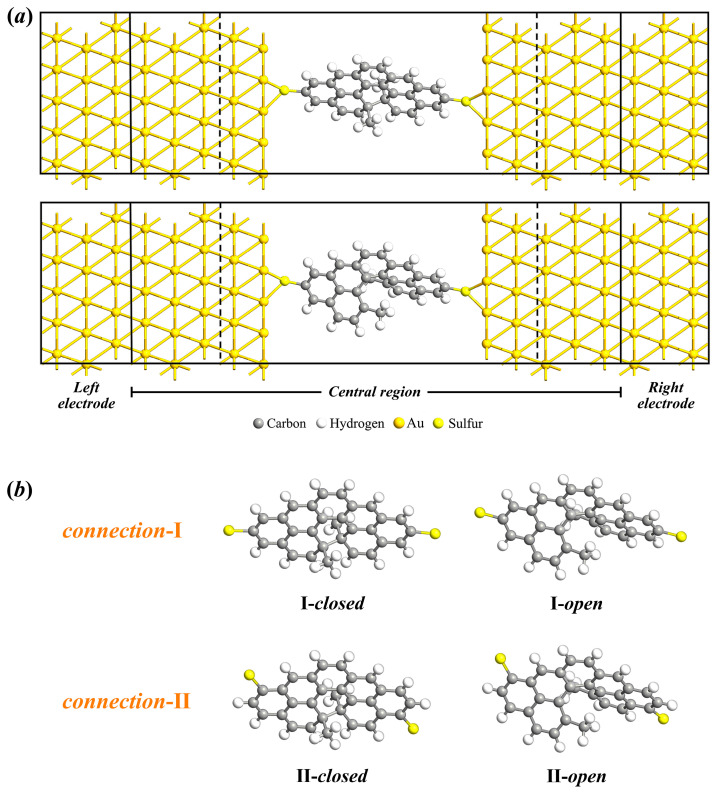
A schematic diagram of (**a**) molecular junctions based on 13,14-dimethylcethrene with Au(111) electrodes. Each junction is composed of three distinct components, i.e., the left electrode, the central scattering region, and the right electrode. (**b**) Forty-five-degree top views of ring-*closed* and ring-*open* isomers with different anchor attachment sites denoted as I(II)-*closed* and I(II)-*open*, respectively.

**Figure 3:**
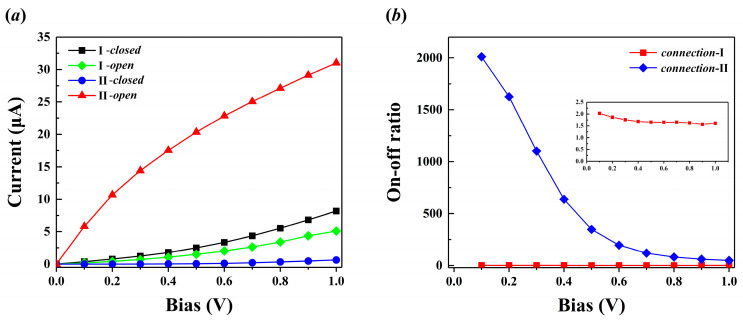
(**a**) The current–voltage (I–V) curves of the molecular junctions based on 13,14-dimethylcethrene with different molecule–electrode interfaces. The solid black and green lines represent the I–V curves of the closed and open configurations with connection I. The solid blue and red lines represent the I–V curves of the closed and open configurations with connection II. (**b**) The *on–off* ratio curves of the molecular junctions. The solid red and blue lines represent the *on–off* ratio curves of the I-configuration and II-configuration, respectively. The inset in the top right corner presents an enlarged depiction of the *on–off* ratio curve for the I-configuration.

As depicted schematically in Figure 2, molecule junctions are formed by the ring-*closed* and ring-*open* isomers of 13,14-dimethylcethrene molecules embedded between two 5 × 5 Au(111) electrodes with distinct molecule–electrode interfaces, denoted as connection I and connection II for clarity. Each junction can be categorized into three parts, i.e., the left electrode, the central scattering region, and the right electrode. In order to gain a comprehensive understanding of the electronic transport characteristics within molecular junctions, rigorous self-consistent calculations were performed. Current–voltage (I–V) curves of molecular junctions consisting of ring-*closed* and ring-*open* isomers with diverse anchor attachment sites are presented in Figure 3a. This figure demonstrates a positive correlation between the current and voltage within the specified bias range of [0.00 V, 1.00 V]. For the molecular junctions consisting of connection I, the current flowing through the closed configuration is greater than that in the open counterpart. The observed variation in electronic transport capacity is consistent with the transport characteristics of most molecular photoswitches based on the electrocyclic reaction, where the closed configuration typically exhibits a strong electronic transport capacity compared to the open configuration. In detail, when a dimethylcethrene molecule undergoes electrocyclization and is converted from the open form to the closed form, the molecular junction simultaneously switches from the *off*-state (low conductance) to the *on*-state (high conductance). The I–V curves of configurations with connection II depict the opposite transport characteristic, where the current value of the open configuration is much greater than that of the closed configuration, indicating a high-conductance state for the open configuration and a low-conductance state for the closed configuration. There is a significant disparity in the currents of the two isomers, and a fascinating switching effect is observed in the configurations of both connections. Here, the *on–off* ratio under the given bias is defined as

(1) $$R(V)=I_{\left(\mathrm{high}\right)}/I_{\left(\mathrm{low}\right)}$$

where *I*_(*high*)_ and *I*_(*low*)_ are the higher and lower current values of the closed and open configurations with the same anchor attachment sites. As depicted in Figure 3b, the *on–off* ratios for two connections show different reductions with an increase in bias voltage. The maximum *on–off* ratio of the II-configuration can reach 2011 (0.10 V) and gradually decreases to 49 (1.00 V), while it is stable at 1.6 in the I-configuration. Overall, the molecular junctions we designed exhibit excellent switching behavior in both configurations, which confirmed the application potential of 13,14-dimethylcethrene as a key component in photoswitchable nanodevices at the single-molecule level. Moreover, the variation attributed to the molecular chirality and the inherent asymmetry of the devices in the electrical transport capacity under positive and negative bias is evidenced in Figure S1.

### 2.2. Bias-Dependent Transmission Spectra

**Figure 4:**
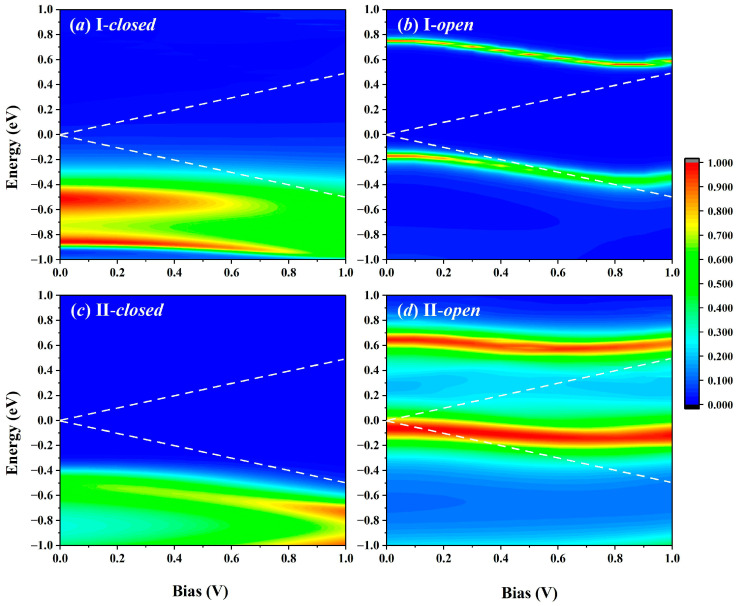
The bias-dependent transmission spectra of the (**a**) closed and (**b**) open configurations consist of connection I. (**c**,**d**) The same case consists of connection II. The white dotted line indicates the bias window.

The bias-dependent transmission spectra (Figure 4) show more details on the electronic transport. A significant transmission peak in the open configuration of I and II is observed at the energy position of −0.12 eV, with the bias increase within the range of [0.00 V, 1.00 V], which moves closer to the Fermi level. As is well known, the current value is obtained according to the Landauer–Büttiker formula [45]. The transmission peak of the I-*open* configuration is narrower than that of the II-*open* configuration and enters the bias window later. Hence, the II-*open* configuration shows the most significant current value at the same bias. In contrast, with I-*closed*, the transmission peak of the II-*closed* configuration is relatively far from the Fermi level (below −0.40 eV), indicating the lower current value at the same bias in four configurations. The combination of the transmission peak located in the vicinity of the Fermi level and the broadened transmission peak near 0.65 eV results in an extremely high current value of the II-*open* configuration.

### 2.3. Bias-Dependent Projected Density of States (PDOS) Spectra

**Figure 5:**
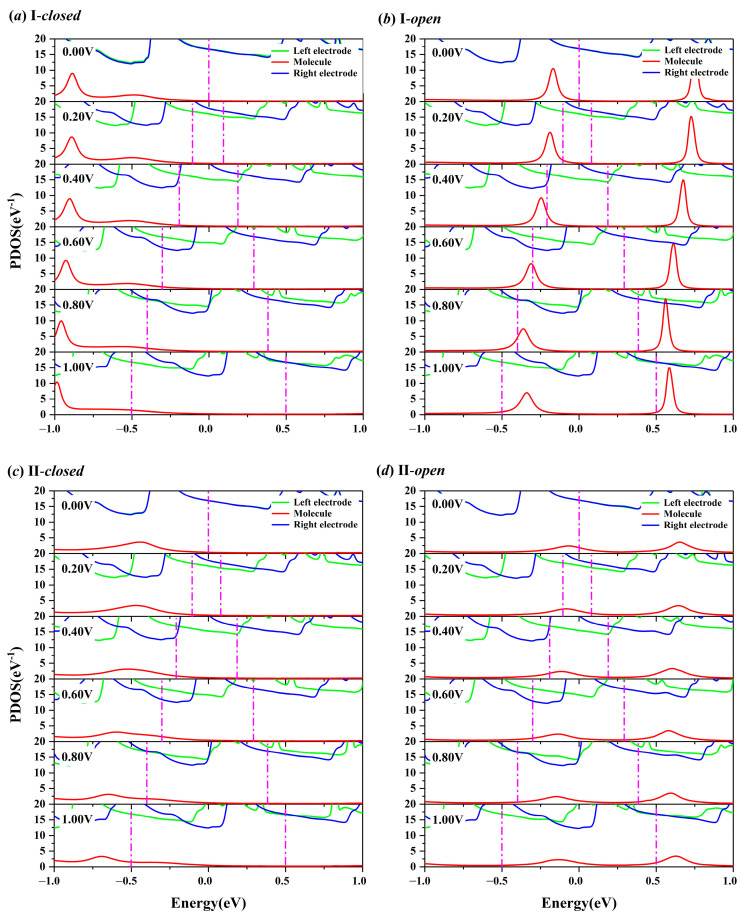
The bias-dependent projected density of states (PDOS) spectra of the (**a**) closed and (**b**) open configurations consists of connection I. (**c**,**d**) The same case consists of connection II. The projection subspace is segmented into three parts, i.e., the left electrode (green line), the molecule (red line), and the right electrode (blue line). The magenta line indicates the bias window.

To elucidate the distinct transport characteristics observed in the four configurations, the PDOS spectra that have been extensively utilized in theoretical analysis were calculated and plotted in Figure 5. They are commonly employed for clear visualization of the contributions from specified atoms to the density of states, thereby facilitating a profound comprehension of electronic transport behavior under various biases. The project subspace of the scattering region is segmented into three parts, i.e., the left electrode, the molecule, and the right electrode. The PDOS peaks contributed from the left and right electrodes for the given bias show notable similarities across all configurations. The PDOS spectra of the left and right electrodes at zero bias overlap. As the bias increases within the range of [0.00 V, 1.00 V], the PDOS spectrum of the left electrode undergoes a systematic migration towards the negative-energy region, and the PDOS spectrum of the right electrode shifts in the opposite direction, reflecting the modulation of electronic states under the influence of the applied potential. It is crucial to emphasize that the effective contribution to the overall electronic transport is exclusively derived from the overlapping regions among the three parts. Obviously, the overlap among the three parts is governed by the part of the molecule, which includes the sulfur atom and the molecule. Although the PDOS spectra of the molecules display diverse responses to the bias voltage, the overlap area of all configurations increases as the bias window widens, indicating a direct correlation between the bias window width and the extent of the overlap. This is in line with the positive correlation between the electronic transport capacity and the bias voltage indicated in Figure 3a.

### 2.4. View of Molecular Energy Levels

**Figure 6:**
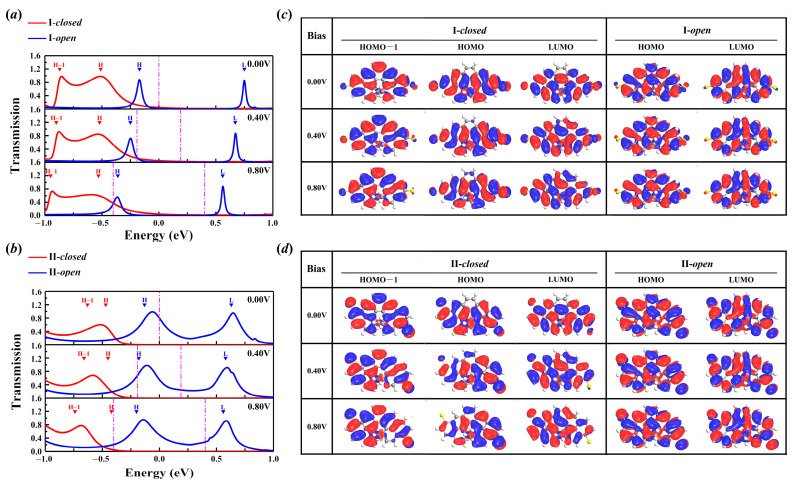
The zero-bias transmission spectra of the (**a**) I-configuration and (**c**) II-configuration based on 13,14-dimethylcethrene. The solid red and blue lines represent the transmission spectra of the closed and open configurations, respectively. The Fermi level is set to zero, and the magenta line indicates the bias window. The red and blue solid down-pointing triangles denote the MPSH eigenvalues of the *closed* and *open* molecules. H and L denote HOMO and LUMO. (**b**,**d**) The bias-dependent spatial distribution of the frontier molecular orbitals in the I-configuration and II-configuration. The isovalue is set to 0.035 for all plots.

The molecules bridging the left and right electrodes play key roles in the electronic structures of the junctions, under the collaborative influence of the adjustment of the molecule–electrode interfaces. So, we calculate the molecular energy levels of 13,14-dimethylcethrene molecules and their thiol-substituted derivatives, as listed in Table 1a. The highest occupied molecular orbitals (HOMOs) of all isomers are closer to the Fermi level than the lowest unoccupied molecular orbitals (LUMOs). The HOMO-LUMO energy gap of the ring-*open* isomer of 13,14-dimethylcethrene is larger than that of the ring-*closed* isomer. In contrast, the HOMO and LUMO of the derivative molecules, influenced by the substituent groups, are near to the Fermi level, resulting in a reduced HOMO-LUMO energy gap. The HOMO-LUMO energy gap of the I-*open* isomer is least affected by the terminal group (<0.02 eV), while the gap of the I-*closed* isomer, which is twice that of II-*closed* isomer, is the most affected (approximately 0.12 eV). For the II-*open* isomer, the HOMO-LUMO energy gap decreases by 0.10 eV. This suggests that the substitution sites on the molecules exert differential effects on their electronic structures. Notably, there is a discrepancy between the molecular energy levels and the distribution of the zero-bias transmission peaks shown in Figure 6. This is attributable to the fact that, in nanodevices, there are broadening molecular energy levels caused by the molecule–electrode coupling.

### 2.5. Molecular Projected Self-Consistent Hamiltonian (MPSH) Analysis

It is widely recognized that the eigenvalues of the molecular orbitals may be significantly affected by interactions between the molecules and the electrodes. The position of the transmission peaks is correlated with the distance of the molecular orbitals from the Fermi level, and the magnitude is governed by the delocalization degree of these orbitals. In this study, for the purpose of revealing the origin of transmission peaks, bias-dependent MPSH analysis was performed. The MPSH of the whole configuration was projected onto the central molecule, including the sulfur atom, to ensure careful consideration of molecule–electrode interaction. The MPSH eigenvalues under a bias voltage of 0.00 V, 0.40 V, and 0.80 V are listed in Table 1b,c and labeled in Figure 6a,b with solid down-pointing triangles. The corresponding spatial distribution of the frontier molecular orbitals is graphically depicted in Figure 6c. Obviously, HOMO is the dominating transport channel in both I-*closed* and II-*closed*. Within the energy range of [−1.00 eV, 1.00 eV], the MPSH eigenvalues in I-*closed* correspond to the positions of the transmission peaks. A broad peak (located around—0.51 eV) originating from the delocalized HOMO state is observed. For II-*closed*, the HOMO state is delocalized at 0.00 V and becomes localized towards the right side of the molecule as the bias voltage increases, finally manifesting as a partially delocalized state and resulting in a limited contribution to charge transport. This is owing to the relatively strong molecule–electrode interaction, which is also confirmed by the discrepancy between the eigenvalues and the position of the transmission peaks in I-*open*. Furthermore, the distance differences in HOMO between I-*closed* and II-*closed* are not big, at 0.04 eV (0.00 V), 0.07 eV (0.40 V), and 0.09 eV (0.80 V). While the observed difference exhibits a positive correlation with the bias voltage, its impact on charge transport is minimal due to its value of less than 0.10 eV. Overall, the HOMO of II-*closed* is more localized than that of I-*closed*, meaning II-*closed* has poorer conductivity. In I-*open*, the HOMO state distributed on the left sulfur atom shifts to the right one, leading to a diminished tunneling effect. On the contrary, the efficient transport channel of II-*open* contributed by the delocalized HOMO and LUMO states grants it powerful electronic transport capability.

The modification of electrodes is revealed by a comparative analysis of the molecular energy levels and the MPSH eigenvalues. Herein, |Δ*Gap*| = |*Gap_isolated_* − *Gap_eigenvalue_*| is calculated in Table 1b,c. Despite the variations observed in the HOMO and LUMO energy levels, the |Δ*Gap*| of I-*closed* and I-*open* is only 0.01 eV, and the |Δ*Gap*|s of II-*closed* and II-*open* are 0.03 eV and 0.08 eV. The HOMO-LUMO energy gap in both the II-*closed* and II-*open* configurations is primarily affected by the modulation of the electrodes.

## 3. Theoretical Methods and Computational Details

Geometric optimization of the molecules and molecular junctions, and subsequent calculations of the electronic transport property, were performed using the Atomistix ToolKit software package [46,47,48]. The Perdew–Burke–Ernzerhof (PBE) functional [49] within the generalized gradient approximation (GGA) was employed to model the exchange-correlation energy. A single-ζ plus polarization (SZP) basis set was utilized for Au atoms, while a double-ζ plus polarization (DZP) basis set was used for non-metal atoms. Core electrons were described using norm-conserving Troullier–Martins pseudopotentials [50]. The Brillouin zone (BZ) was sampled with a 4 × 4 × 150 *k*-point grid using the Monkhorst–Pack method. The convergence criterion and the energy cutoff were, respectively, set to 10^−5^ eV and 150 Ry to ensure calculation accuracy.

Under a specified bias voltage *V_b_*, the tunneling current through molecular junctions was calculated using the Landauer–Büttiker formula [45]:


(2) $$I(V)=\frac{2e}{h}\int T(E,V_{b})[f_{L}(E-\mu_{L})-f_{R}(E-\mu_{R})]dE$$

Here, *T*(*E*,*V_b_*) is the electronic transmission coefficient of the incident energy *E*, *μ*_*L*(*R*)_ represents the electrochemical potential of the left (right) electrode, and *f*_*L*(*R*)_(*E* − *μ*_*L*(*R*)_) is the Fermi–Dirac distribution function of the left (right) electrode.

## 4. Conclusions

In summary, we have proposed and simulated molecular junctions based on 13,14-dimethylcethrene sandwiched between two Au(111) electrodes. We find that 13,14-dimethylcethrene is a promising molecular photoswitch in which the HOMO-LUMO energy gap of the ring-*open* isomer is smaller than that of the ring-*closed* isomer. The maximum *on–off* ratio of the dimethylcethrene-based photoswitch can reach as high as 10^3^. It has been determined that the molecule–electrode interface properties have a critical impact on the transport behavior of the nanodevices. These findings unravel potential insights for designing molecular nanodevices based on cethrene at the single-molecule level.

## Figures and Tables

**Table 1:** (**a**) Energy levels of isolated molecules and (**b**,**c**) MPSH eigenvalues in different configurations.

(a)	Configurations	Ring-*Closed*	Ring-*Open*	I-*Closed*	I-*Open*	II-*Closed*	II-*Open*
	LUMO+1 (eV)	1.20	1.62	1.10	1.64	1.14	1.61
	LUMO (eV)	1.01	0.47	0.95	0.46	0.98	0.42
	HOMO (eV)	−1.01	−0.47	−0.95	−0.46	−0.98	−0.42
	HOMO−1 (eV)	−1.27	−1.78	−1.35	−1.36	−1.16	−1.63
	Gap (eV)	2.02	0.94	1.90	0.92	1.95	0.84
**(b)**	**Configurations**	**I-*closed***			**I-*open***		
	Bias (V)	0.00	0.40	0.80	0.00	0.40	0.80
	LUMO+1 (eV)	1.60	1.60	1.58	1.94	1.85	1.73
	LUMO (eV)	1.40	1.37	1.30	0.75	0.67	0.56
	HOMO (eV)	−0.51	−0.52	−0.53	−0.17	−0.25	−0.36
	HOMO−1 (eV)	−0.87	−0.90	−0.95	−1.38	−1.37	−1.36
	*Gap* (eV)	1.91	1.89	1.83	0.92	0.92	0.92
	|Δ*Gap*| (eV)	0.01	-	-	0.01	-	-
**(c)**	**Configurations**	**II-*closed***			**II-*open***		
	Bias (V)	0.00	0.40	0.80	0.00	0.40	0.80
	LUMO+1 (eV)	1.65	1.65	1.65	1.76	1.70	1.67
	LUMO (eV)	1.45	1.42	1.33	0.63	0.58	0.56
	HOMO (eV)	−0.47	−0.45	−0.42	−0.13	−0.18	−0.20
	HOMO−1 (eV)	−0.63	−0.66	−0.74	−1.51	−1.53	−1.52
	*Gap* (eV)	1.92	1.87	1.75	0.76	0.76	0.76
	|Δ*Gap*| (eV)	0.03	-	-	0.08	-	-

## Data Availability

The original contributions presented in the study are included in the article and Supplementary Material, further inquiries can be directed to the corresponding authors.

## Supplementary Materials

The following supporting information can be downloaded at: https://www.mdpi.com/article/10.3390/molecules29204912/s1, Figure S1: The current–voltage (I–V) curves of the molecular junctions within [−1.00 V, 1.00 V].
