# Supplementary material for: Rational Design of High-Performance Photocontrolled Molecular Switches Based on Chiroptical Dimethylcethrene: A Theoretical Study
Source: Molecules. 2024 Oct 17;29(20):4912. doi: 10.3390/molecules29204912 (PMC11510373; doi:10.3390/molecules29204912)
Supplement: Supplementary file 1 [file molecules-29-04912-s001.zip › molecules-3250309-supplementary.pdf]

Rational Design of High-Performance Photocontrolled Molecular Switches Based on Chiroptical Dimethylcethrene: A Theoretical Study

Li Han,[a,b] Mei Wang,[c] Yifan Zhang,[b] Bin Cui [a]* and Desheng Liu [a]*

[a] *School of Physics, Shandong University, Jinan 250100, China*

[b] *School of Physics and Electronic Engineering, Jining University, Qufu 273155, China*

[c] *School of Science, Shandong Jiaotong University, Jinan 250357, China*

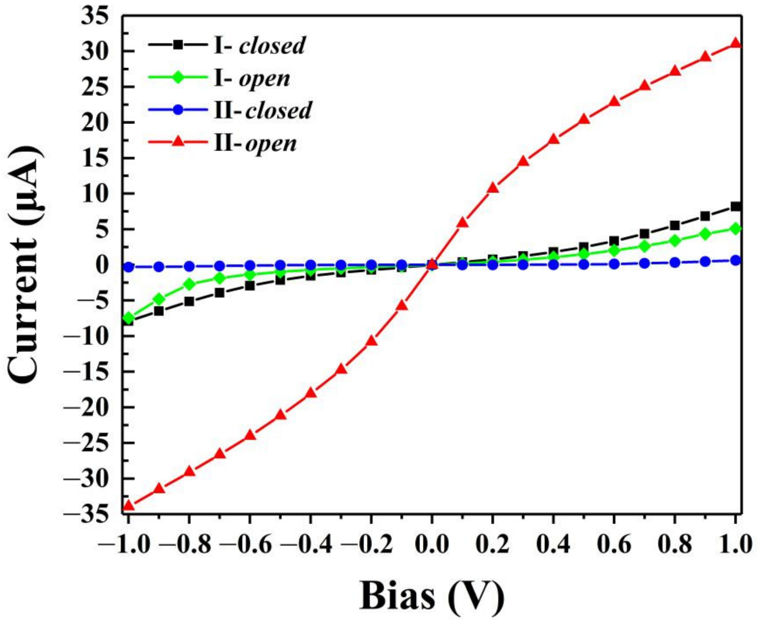

Figure S1. The current-voltage (I-V) curves of the molecular junctions within [-1.00 V, 1.00 V]. The solid black and green lines represent the I-V curves of the closed and open configurations with connection I. The solid blue and red lines represent the I-V curves of the closed and open configurations with connection II.
